# Optimization of methylene blue removal by stable emulsified liquid membrane using Plackett–Burman and Box–Behnken designs of experiments

**DOI:** 10.1098/rsos.171220

**Published:** 2018-02-14

**Authors:** Meriem Djenouhat, Farida Bendebane, Lynda Bahloul, Mohamed E. H. Samar, Fadhel Ismail

**Affiliations:** 1 LOMOP Research Laboratory, Badji Mokhtar University of Annaba, Annaba, Algeria; 2 Process Engineering Department, Badji Mokhtar University of Annaba, Annaba, Algeria; 3 Chemistry Department, Badji Mokhtar University of Annaba, Annaba, Algeria; 4 Welding and NDT Research Center (CSC), BP 64 Cheraga, Algeria

**Keywords:** emulsified liquid membrane, extraction, methylene blue, modelling, optimization

## Abstract

The stability of an emulsified liquid membrane composed of Span80 as a surfactant, D2EHPA as an extractant and sulfuric acid as an internal phase was first studied according to different diluents and many operating parameters using the Plackett–Burman design of experiments. Then the removal of methylene blue from an aqueous solution has been carried out using this emulsified liquid membrane at its stability conditions. The effects of operating parameters were analysed from the Box–Behnken design of experiments. The optimization of the extraction has been realized applying the response surface methodology and the results showed that the dye extraction yielding 98.72% was achieved at optimized conditions.

## 1. Introduction

The industrial revolution has beneficial results for humans, facilitating and simplifying life. Since the end of the last century, manufacturers have showed an interest in raising and improving their production without environmental damages caused by unauthorized discharges taking into account the norms suggested by the World Health Organization and environmental organizations. Water, which occupies more than 70% of the Earth's surface and is the life source of living creatures, is affected by pollutants from diverse nature and complexes.

Principally, pollutants may be organic contaminants or heavy metals. Their presence in industrial effluents or clean drinking water may cause problems in public health by reason of their absorption and then their accumulation in humans. The regulations concerning water pollution require textile industries to reduce the amount of dyes in their discharges [1].

Dye industries are nowadays an important section of chemistry; world production is estimated at more than 800 000 tons per year of which 140 000 tons are lost with effluents during processes of different application steps and procedures [2]. China and countries of Maghreb are the biggest producers of textile industries, fur and leather dyes are also used in industries of plastic materials, ceramic, printing, pharmaceutical and cosmetic industries, rather than agro-food industries [3].

Methylene blue (MB), known as methylthioninium chloride, is one of the basic dyes strongly used in various fields as a redox indicator or histological dye. It is also used as a vital dye of living cells, for colouring cotton, wood, silk and paper [4]. It is an optical limiter combined with polymer, for eye protection against intense lasers and a good antiseptic for internal and external use [5,6]. But, it can cause eye burns responsible for permanent injury to human and animal eyes [4].

The treatment for industrial effluents containing this type of dye is of great interest for many technologies. The discoloration of these effluents has been achieved by different processes such as coagulation–flocculation, membrane filtration, adsorption, chemical oxidation, biological processes and liquid–liquid extraction [7]. However, some of these treatments present disadvantages mostly if the treated water is from agricultural fields because the products of degradation from biological treatment are unknown or toxic products. The extraction by emulsion liquid membrane (ELM) is an alternative method to liquid–liquid extraction. Water in oil (W/O) and oil in water (O/W) emulsions were employed for the first time in different main industrial domains such as the energy agro-food industries (such as vinaigrette, butter, mayonnaise and ice cream), cosmetics (such as moisturizing cream and body lotion), polymers (such as polymerization in emulsion and latex) or aqueous paint [8]. Also the use of emulsions has been widened for the recovery and concentration of heavy metals such as double emulsion W/O/W or O/W/O, named ELM which was first used in hydrocarbon separation [9], then in elimination of soluble constituents (phenol, phosphoric acid, sodium nitrate and ammonia) from aqueous solutions [10] and since 1983 the ELM has been applied in industry. The ELM presents several advantages such as high transport speed, high contact area, high flow, possibility of total recovery of constituents and no production of sludge in environmental technology [11]. Furthermore, the inopportune breakage of emulsion droplets, the difficulties limited to phases separation and the impossibility of acceding the receive phase for sample or regulation make this technique difficult for industrial application. In the ELM, diluent represents more than 80% of components. So, it should have a low volatility, a low solubility in water and a high interfacial tension with water (water-diluent) [12].

In order to create stable emulsions for extraction of dyes, Plackett–Burman designs were carried out with the application of different diluents such as kerosene, gas oil, *n*-heptane and cyclohexane. For this, seven factors were developed: emulsification time, concentration of surfactant (Span80), concentration of internal phase (H_2_SO_4_), concentration of extractant (D2EHPA), agitation speed, volume ratio of internal phase to organic phase (A/O) and volume ratio of external phase to emulsion phase (*V*_ext_/*V*_emul_).

In the second step, an extraction of MB with the application of response surface methodology ‘Box–Behnken design' has been realized by the employment of the system 'Span80–D2EHPA–kerosene', which gave the most stable emulsions.

## 2. Experimental

### 2.1. Reagents

The reagent used in emulsion stability was Span80 (monooleate of sorbitan), an anionic commercial surfactant. It is a viscous yellow liquid with a hydrophilic/lipophilic balance of 4.3 and a molecular weight of 428.6 g mol^−1^. A mobile transporter of dyes, D2EHPA (di-2-ethylhexylphosphoric acid), a colourless and viscous liquid, is very soluble in organic solvents. Compounds, surfactants and extractants were provided by Sigma-Aldrich (USA). Kerosene, gas oil, heptanes, cyclohexane are the different diluents in this study. Commercial kerosene (density 800 kg m^−3^ at 25°C) is a complex mixture of 35% of alkanes, 15% of aromatic compounds and 60% of cycloalkanes (naphthenes), used as a civil or army airplane fuel [13], which is a product of Sigma-Aldrich (Netherlands). Heptane, a volatile liquid, with characteristic odour, is insoluble in water but miscible with various organic solvents. Cyclohexane, a colourless and mobile liquid, with acrid odour, has a very low solubility with water (58 mg l^−1^ at 25°C), miscible in different organic solvents which is purchased from Sigma-Aldrich (Germany). According to the elementary analyses, gas oil, which is classed as a combustible liquid named fuel oil and made from crude petroleum, comprises carbon, hydrogen and heteroatom (OSN) [14]. The fuel oil used in this work is the Algerian product. Sulfuric acid (Fulka) with a molecular weight of 98.08 g mol^−1^ and a density of 1.84 (at concentration of 95–97%) was used in the internal aqueous phase as dye-acceptor stripping agent. Demineralized water (pH = 5.0–6.5) was used as an external aqueous phase. MB (cationic dye), with chemical formula C_16_H_18_N_3_ClS and molecular weight 319.86 g mol^−1^, was taken as the pollutant model and provided by Sigma-Aldrich. In order to generate a stable emulsion for extraction, homogenizer-type Moulinex active flow technology, with a capacity of 700 W has been employed. Then a mechanical stirrer-type RW20 Kjank & Kunkel was used for dispersing emulsified membrane into a beaker containing the solution external phase (initial concentration of MB).

### 2.2. Emulsion preparation

First, the organic phase was prepared by dissolving a suitable amount of D2EHPA and Span80 in different diluents under a gentle mixing by a magnetic stirrer. In order to prepare the W/O emulsion, the aqueous stripping solutions of sulfuric acid (H_2_SO_4_) were dispersed in the organic phase by using a high-speed homogenizer with maximal power during 1–6 min. The W/O/W double emulsions were obtained by contacting the first W/O emulsion with the external phase; a magnetic agitator was used for this and fixed at an agitation speed of 100–400 r.p.m. After 10 min of contact time, the pH of external phase decreases because of the leakage of H^+^ ions in the external phase. This phenomenon-nominated emulsion brakeage (*B*) was determined by the application of a balance material vis-a-vis ions H^+^ (equation (2.1)) [15]. The pH values of the external phase solutions were set by pH meter (WIW inoLab PH 7110).

2.1 $$B=V_{\mathrm{ex}}\times \frac{\left\{[{\text{H}}^{+}{\text{] }}_{\mathrm{ex}}-{\left[{\text{H}}^{+}\right]}_{i}\right\}}{\left\{V_{i}\times {\left[{\text{H}}^{+}\right]}_{0}\right\}}\times 100,$$

where *B* is the break-up per cent of the emulsion which represents the stability of the ELM system, [H^+^]_0_ is the initial concentration of total H ions in the internal aqueous phase, [H^+^]_ex_ is the concentration of H ions in the external aqueous phase at contact time, [H^+^]_i_ is the initial concentration of total H ions in the external aqueous phase and *V*_ex_ and *V*_i_ are the volume of the external and the internal aqueous phase, respectively.

After optimization of stability parameters, the extraction was realized by contacting 130 ml of MB solutions (10, 30 and 50 mg l^−1^) with 26 ml of emulsions. The samples preceded were dosed after 15 min within optimized time, and absorbance measurements were carried out using a PRIM-SECOMAM (UV/VIS) spectrophotometer. The maximum wavelength of absorption is 664 nm obtained experimentally and used for all samples of residual MB. The extracted quantity of MB was calculated using the following equation:

2.2 $$Y(\%)=\left[1-\left[\frac{C_{f\;\mathrm{ext}}\times V_{f\;\mathrm{ext}}}{C_{0\mathrm{ext}}\times V_{0\mathrm{ext}}}\right]\right]\times 100.$$

*V*_0ext_ is the initial volume of the external phase; *V*_f ext_ is the final volume of the external phase; *C*_0ext_ is the initial concentration of MB; C_f ext_ is the final concentration of MB.

## 3. Results and discussion

### 3.1. Stability study

#### 3.1.1. Experimental results

With the objective to determine the important factors, the statistical software Minitab-16 [16] was used and a Plackett–Burman design was applied. The influence of experimental parameters on W/O/W emulsion stability has been studied with seven factors and four diluents. Results are regrouped in tables 1 and 2.

**Table 1.** Factors and levels.

		level	
factors	unit	low (−1)	high (+1)
emulsification time (time)	min	1	6
agitation speed (SV)	r.p.m.	100	400
extractant (D2EHPA)	% by weight	5	15
surfactant (Span80)	% by weight	1	8
volume ratio of external phase to emulsion phase (*V*_ext_/*V*_emul_)	—	2	6
volume ratio of aqueous phase to organic phase (A/O)	—	0.9	1.85
volume ratio of aqueous phase to organic phase (A/O) (for *n*-heptane)	—	0.8	1.60

**Table 2.** Experimental results according to Plackett–Burman design.

std order	time	SV	D2EHPA	Span80	A/O	V_ex_/V_em_	[H_2_SO_4_]int	Bk (%)	Bg (%)	Bh (%)	Bc (%)
20	1	100	15	8	1.85 (1.60)	2	2	0.18	0.08	0.12	0.58
18	6	400	15	1	1.85 (1.60)	6	0.05	7.71	520.20	98.64	173.76
1	6	100	15	1	0.9 (0.8)	2	2	7.84	25.53	33.43	6.43
13	6	100	15	1	0.9 (0.8)	2	2	6.28	28.78	35.49	5.75
19	1	400	15	8	0.9 (0.8)	6	2	1.96	1.38	2.21	1.89
5	6	400	5	8	1.85 (1.60)	2	2	1.30	1.01	9.31	4.20
17	6	400	5	8	1.85 (1.60)	2	2	0.86	4.14	2.38	3.57
7	1	400	15	8	0.9 (0.8)	6	2	1.51	1.37	3.31	1.22
10	6	100	5	1	1.85 (1.60)	6	2	2.58	34.53	2.63	3.70
14	6	400	5	8	0.9 (0.8)	2	0.05	17.62	49.85	5.74	81.22
2	6	400	5	8	0.9 (0.8)	2	0.05	15.13	48.23	6.53	82.33
4	6	100	15	8	0.9 (0.8)	6	0.05	10.38	4.79	3.59	5.58
24	1	100	5	1	0.9 (0.8)	2	0.05	18.18	44.98	2.69	6.15
3	1	400	15	1	1.85 (1.60)	2	0.05	41.88	82.98	19.38	32.62
11	1	400	5	1	0.9 (0.8)	6	2	2.96	37.08	7.23	5.49
22	6	100	5	1	1.85 (1.60)	6	2	3.31	33.98	2.67	3.68
21	1	100	5	8	1.85 (1.60)	6	0.05	5.27	2.19	1.31	0.72
12	1	100	5	1	0.9 (0.8)	2	0.05	11.24	39.46	3.69	5.61
16	6	100	15	8	0.9 (0.8)	6	0.05	11.70	4.10	3.23	5.07
15	1	400	15	1	1.85 (1.60)	2	0.05	27.93	86.28	24.97	32.25
6	6	400	15	1	1.85 (1.60)	6	0.05	68.22	520.20	94.63	171.39
23	1	400	5	1	0.9 (0.8)	6	2	2.,95	33.74	8.26	5.37
9	1	100	5	8	1.85 (1.60)	6	0.05	5.64	5.45	0.97	0.55
8	1	100	15	8	1.85 (1.60)	2	2	0.17	0.19	0.15	0.69

#### 3.1.2. Pareto chart

**Figure 1.**
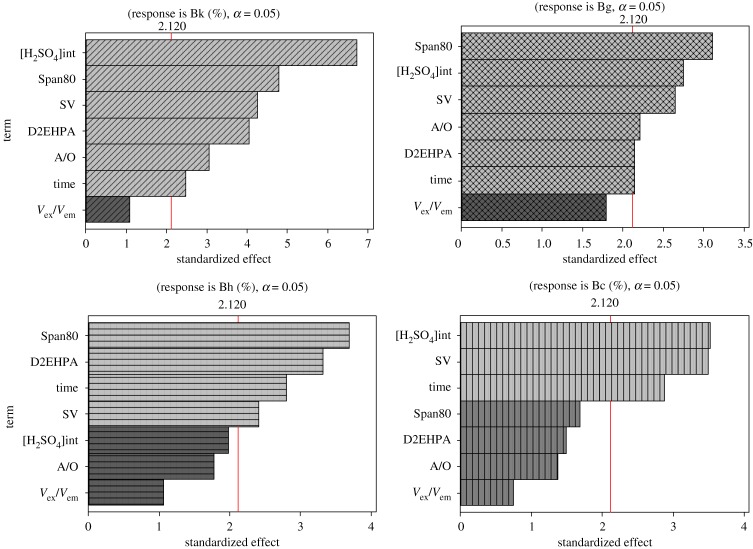
Pareto charts of effects for kerosene, gas oil, heptane and cyclohexane.

The Pareto charts of effects for different diluents such as kerosene, gas oil, *n*-heptane and cyclohexane are shown in figure 1.

Diluent, an important element in ELM, is a major constituent limited for membrane to about 90%; for this reason, the stability of emulsions is effectuated by varying for diluents such as kerosene, gas oil, *n*-heptane and cyclohexane in order to offer better stability and good mass transfer.

According to Pareto charts, the kerosene as a multi-component fuel gives stable emulsions in this study field, with six significant factors out of seven which are the concentration of internal phase, the concentration of surfactant, the stirring speed, the concentration of extractant, the A/O ratio and the emulsification time. The *V*_ext_/*V*_emul_ ratio is the only nonsignificant factor. Similarly gas oil, which is a viscous fuel, insoluble in water, has six main factors; but, it gives emulsions less stable than kerosene.

*n*-Heptane presents only four significant factors as follows: the concentration of surfactant, the concentration of extractant, the emulsification and stirring speed.

Therefore, cyclohexane is a good solvent for extraction of non-polar organic molecules [17] with only three main factors (the concentration of internal phase, the stirring speed and the emulsification time).

Arrangement of these factors for each diluent depends on several phenomena; unlike volatility, viscosity is a very important parameter in the formation of emulsions.

The kerosene and gas oil are both essential oils from petroleum distillation at high temperature, usually containing 10 to 22 to 16 carbon atoms per molecule and 12–22 carbon per molecules, respectively [12]. These two diluents have the same characteristics, although gas oil has higher carbon number and dynamic viscosity than kerosene. So, these solvents have six identical main factors, starting with the surfactant which is an essential factor because the emulsions are unstable thermodynamic systems, which separate more or less rapidly, in two phases, where the surfactant forms a thin layer around the membrane by adsorbing on the surface, thereby reducing its interfacial tension and enhancing its stability [18]. Second, the concentration of sulfuric acid which is an aqueous solution of greater density than water varies between 1.0069 (1%) and 1.84 (95% of acid), but immiscible with the diluents studies; this immiscibility is essential for the formation of the emulsions. The same classification of the internal phase for kerosene, gas oil and cyclohexane can be explained by the densities which are near and take, respectively, the following values: 0.77, 0.796 and 0.76 (g cm^−3^). Thus, the difference in density between the aqueous phase and the oily phase is almost the same. Then, the stirring speed is a main factor for extraction; its regulation is necessary in order to avoid the rupture of the multiple emulsions and has the same importance for all diluents. By contrast, *n*-heptane as cyclohexane classifies emulsification time among the first three main factors; because liquids which have a low viscosity are relatively volatile, this volatility affects the stability of the emulsions by loss of material.

#### 3.1.3. Main effects

From main effects plots, the concentration of surfactant (Span80) and the concentration of internal phase (H_2_SO_4_) have a negative effect on the emulsion stability with all diluent studies; as 1% of the surfactant is insufficient to form the interfacial layer and the increases in the amount of the surfactant reduce the breakdown of the emulsions [19]. Similarly, for internal phase concentration, the evolution of the concentration leads to an increase in the density difference between the organic and the internal phase, which promotes the formation of the emulsions.

**Figure 2.**
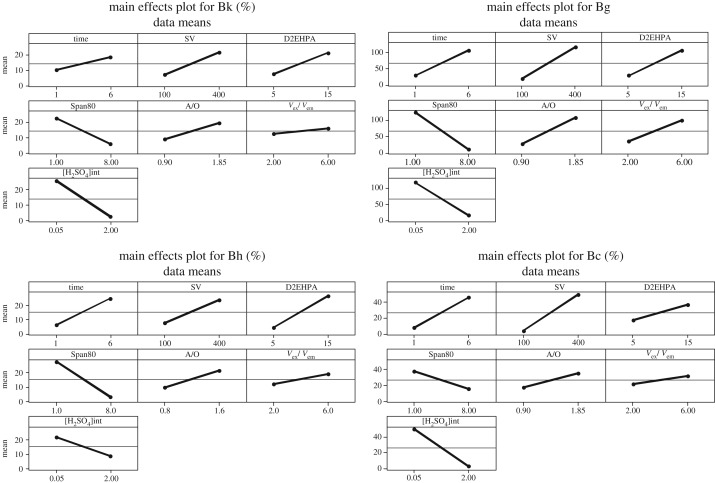
Main effects plots of parameters for kerosene, gas oil, heptane and cyclohexane.

**Figure 3.**
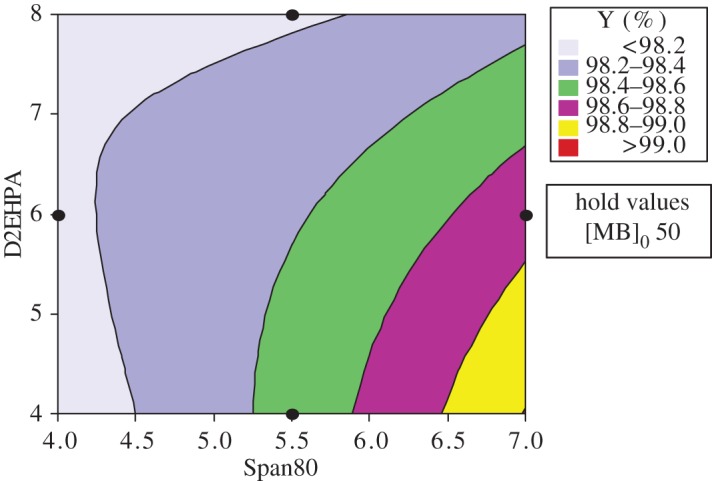
Contour plot of Y versus D2EHPA; Span80 at maximal [MB]_0_.

**Figure 4.**
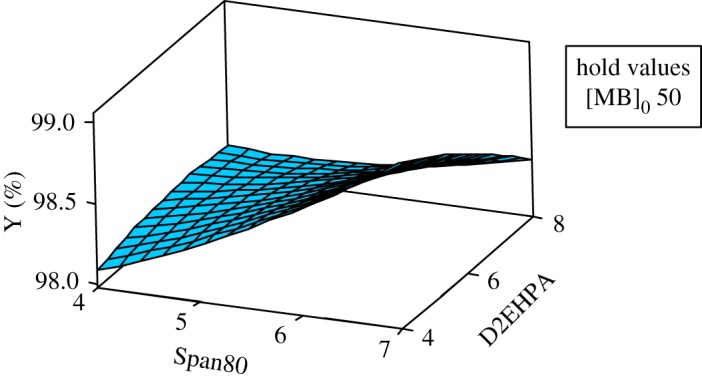
Surface plot of Y(%) versus D2EHPA ; Span80 at maximal [MB]_0_.

**Figure 5.**
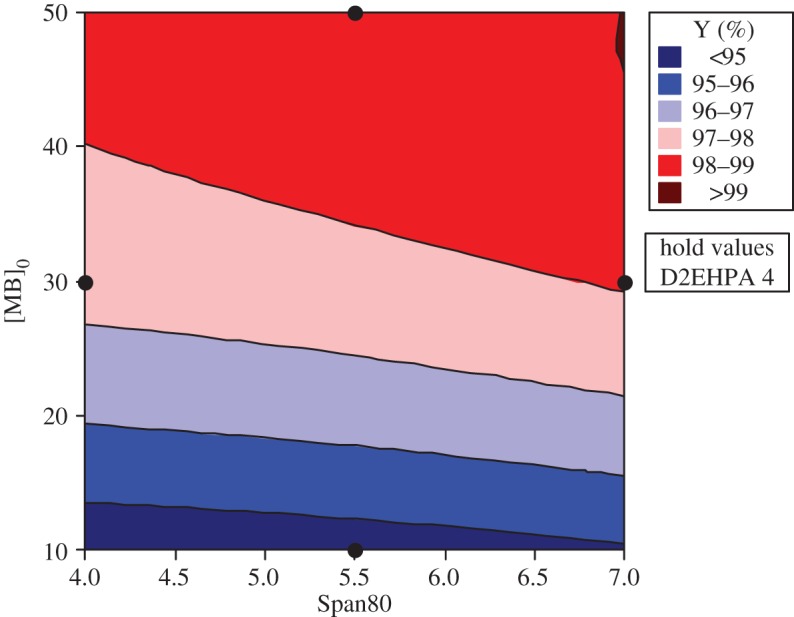
Contour plot of Y versus [MB]_0_; Span80 at minimal of D2EHPA.

**Figure 6.**
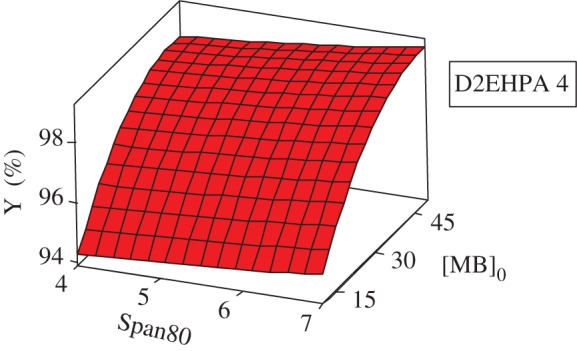
Surface plot of Y(%) versus [BM]_0_; Span80 at minimal of D2EHPA.

The kerosene system presents a more stable system in comparison to other diluents with the minimum breaking rate (figures 2–6). The stirring speed and the D2EHPA concentration have a positive and strong effect; the emulsification time, the O/A ratio have a positive and weak effect on the response. But, *V*_ext_/*V*_emul_ ratio has no effect on the response studied. The increase of the stirring speed causes the shearing of the double emulsions W/O/W, so a moderate stirring speed must be chosen in order to make the extraction successful, the extractant concentration also has a positive effect; by increasing D2EHPA, concentration break-up becomes very important, which may be due to the behaviour of D2EHPA molecules on nonpolar solvents by promoting the formation of dimers in these solvents [20]. The emulsification time has a positive effect on the response, but it is not beneficial to the stability. The ratio A/O also has a positive effect because by increasing this ratio the volume of the internal phase becomes greater than the organic phase; and thereafter the emulsion becomes more concentrated by increasing the viscosity of the mixture, which favours the coalescence and the increase in the size of the dispersed droplets of the internal phase [19,21].

Finally, the *V*_ext_/*V*_emul_ ratio has no effect on the stability, as the emulsion studied is the diluted emulsion, where the interaction between drops of the emulsion and the external phase is negligible [22]. The *n*-heptane is the second stable system with a limit rate of 40%; the main effects of the various factors act in the same way with a positive slope (more or less strong). The breakdown rate of cyclohexane and gas oil is greater compared with that of the first two diluents, but the effects of factors are stronger.

#### 3.1.4. Analysis of variance

The comparison of *p* values (probability *p*) with the significant value chosen in this work at a confidence level of 95% (*α* = 0.05) is summarized in tables 3–7. The latter shows that the analysis of variance applied to experimental data of the system 1 (kerosene– Span80–D2EHPA); having six significant factors has a value less than 0.05; four of them are highly significant and take a value less than or equal to 0.001 which are stirring speed, D2EHPA concentration, Span80 concentration and sulfuric acid concentration; this indicates that the latter have a great influence on the stability of the W/O/W emulsions.

**Table 3.** Effects and coefficients of the estimated yield of kerosene (coded units).

term	effect	coef	SE coef	*t*	*p*
constant	14.24	1.725	1.725	8.26	0.000
time	8.51	4.25	1.725	2.47	0.025
SV	14.69	7.34	1.725	4.26	0.001
D2EHPA	13.98	6.99	1.725	4.05	0.001
Span80	−16.53	−8.27	1.725	−4.79	0.000
A/O	10.52	5.26	1.725	3.05	0.008
*V*_ex_/*V*_em_	3.71	1.86	1.725	1.08	0.297
[H_2_SO_4_]_int_	−23.17	−11.58	1.725	−6.72	0.000

**Table 4.** Analysis of variance for yield of kerosene (coded units).

source	DF	seq SS	adj SS	adj MS	*F*	*p*
main effects	7	8507.65	8507.65	1215.38	17.03	0.000
residual error	16	1142.04	1142.04	71.38		
total	23	9649.69

**Table 5.** Effects and coefficients of the estimated yield of gas oil (coded units).

term	effect	coef	SE coef	*t*	*p*
constant	67.11	18.31		3.67	0.002
time	78.35	39.17	18.31	2.14	0.048
SV	96.87	48.43	18.31	2.65	0.018
D2EHPA	78.44	39.22	18.31	2.14	0.048
Span80	−113.75	−56.87	18.31	−3.11	0.007
A/O	80.99	40.50	18.31	2.21	0.042
*V*_ex_/*V*_em_	65.62	32.81	18.31	1.79	0.092
[H_2_SO_4_]_int_	−100.58	−50.29	18.31	−2.75	0.014

**Table 6.** Effects and coefficients of the estimated yield of *n*-heptane (coded units).

term	effect	coef	SE coef	*t*	*p*
constant	/	15.52	3.332	4.66	0.000
time	18.66	9.33	3.332	2.80	0.013
SV	16.05	8.03	3.332	2.41	0.028
D2EHPA	22.15	11.07	3.332	3.32	0.004
Span80	−24.57	−12.29	3.332	3.69	−0.002
A/O	11.82	5.91	3.332	1.77	0.095
*V*_ex_/*V*_em_	7.07	3.53	3.332	1.06	0.305
[H_2_SO_4_]_int_	−13.18	−6.59	3.332	−1.98	0.065

**Table 7.** Effects and coefficients of the estimated yield of cyclohexane (coded units).

term	effect	coef	SE coef	*t*	*p*
constant	/	26.66	6.568	4.06	0.001
time	37.79	18.90	6.568	2.88	0.011
SV	45.90	22.95	6.568	3.49	0.003
D2EHPA	19.55	9.78	6.568	1.49	0.156
Span80	−22.05	−11.02	6.568	−1.68	0.113
A/O	17.97	8.98	6.568	1.37	0.190
V_ex_/V_em_	9.75	4.87	6.568	0.74	0.469
[H_2_SO_4_]_int_	−46.22	−23.11	6.568	−3.52	0.003

System 2 (Gas oil–Span80–D2EHPA) gives three values close to 0.05 which are emulsification time (*p* = 0.048), D2EHPA concentration (*p* = 0.048) and A/O ratio (*p* = 0.042); internal phase concentration and Span80 concentration have a low value of *p* (table 5), which explains why this system is not stable under these conditions and choice of levels requires research of the appropriate experimental field.

Table 6 shows the values of *p* for system 3 (*n*-heptane–Span80–D2EHPA). The significant factors with a value less than 0.05 are the same ones reported in the Pareto chart, where the surfactant concentration has the lowest value (0.002).

The cyclohexane–Span80–D2EHPA system is classed in the last position with three factors having a value less than 0.05 (table 7).

#### 3.1.5. Polynomial regression

A mathematical function of first or *n* order *y* = *f*(xi) is a function that connects the response Y (rate of rupture) and the various parameters studied xi. The interest of modelling the response is to be able to calculate all the responses of the domain studied without being obliged to make the experiments.

The different mathematical models for kerosene, gas oil, *n*-heptane and cyclohexane are given in equations (3.1), (3.2), (3.3) and (3.4), respectively.

Mathematical model for kerosene stability (uncoded units)

3.1 $$\begin{array}{rl} \text{Bk(}\%\text{) } & \;=-14.0728+1.70108\times (t)+0.0489653\times (\mathrm{SV})\\ & \;\;+1.39753\times (\text{D2EHPA})-2.36166\times (\text{Span}80)\\ & \;\;+11.0785\times \left(A/O\right)+0.928603\times \left(\frac{V_{\mathrm{ex}}}{V_{\mathrm{em}}}\right)-11.8798\times ({\left[{\text{H}}_{2}\text{S}{\text{O}}_{4}\right]}_{\mathrm{int}}\text{) }. \end{array}$$

Mathematical model for gas oil stability (uncoded units)

3.2 $$\begin{array}{rl} \text{Bg}(\%) & \;=-203.758+15.6692\times (\text{time})+0.322885\times (\text{SV})\\ & \;\;+7.84367\times (\text{D2EHPA})-16.2495\times (\text{Span80})\\ & \;\;+85.2569\times \left(A/O\right)+16.4062\times \left(\frac{V_{\mathrm{ex}}}{V_{\mathrm{em}}}\right)-51.5774\times ({\left[{\text{H}}_{2}\text{S}{\text{O}}_{4}\right]}_{\mathrm{int}}). \end{array}$$

Mathematical model for *n*-heptane stability (uncoded units)

3.3 $$\begin{array}{rl} \text{Bh}(\%) & \;=-35.1319+3.73296\times (\text{time})+\;0.0535031\times (\text{SV})\\ & \;\;+2.21462\times (\text{D2EHPA})-3.51012\times (\text{Span80})\\ & \;\;+14.7705\times \left(A/O\right)+1.76685\times \left(\frac{V_{\mathrm{ex}}}{V_{\mathrm{em}}}\right)-6.75951\times ({\left[{\text{H}}_{2}\text{S}{\text{O}}_{4}\right]}_{\mathrm{int}}]). \end{array}$$

Mathematical model for cyclohexane stability (uncoded units)

3.4 $$\begin{array}{rl} \text{Bc}(\%) & \;=-54.8783+7.55861\times (\text{time})+0.152995\times (\text{SV})\\ & \;\;+1.95520\times (\text{D2EHPA})-3.14964\times (\text{Span80})+18.9120\times \left(\left(A/O\right)\right)\\ & \;\;+2.43725\times \left(\frac{V_{\mathrm{ex}}}{V_{\mathrm{em}}}\right)-23.7047\times ({\left[H_{2}SO_{4}\right]}_{\mathrm{int}}). \end{array}$$

### 3.2. Extraction study

#### 3.2.1. Experimental results

The extraction of MB has been realized by the application of the Box–Behnken design; for this, three variables were considered as important process parameters: the concentration of extractant, the concentration of surfactant and the concentration of pollutant phase (dye). Table 8 regroups the factors and studied fields. From the experimental results (table 9), the MB extraction according to response surface methodology gave a yield of 98.72%.

**Table 8.** Experimental range and levels factors in coded and un-coded forms.

		range and levels		
factors		(−1)	(0)	(+1)
Span80	%	4	5.5	7
D2EHPA	%	4	6	8
[MB]_0_	ppm	10	30	50

**Table 9.** Experimental responses of extraction according to Box–Behnken design.

*n*	std order	Span80	D2EHPA (%w)	[MB]_0_ (ppm)	Y (%)	Y_th_ (%)
1	14	5.5	6	30	97.85	97.89
2	12	5.5	8	50	98.20	98.18
3	1	4	4	30	97.36	97.33
4	9	5.5	4	10	94.50	94.53
5	13	5.5	6	30	97.89	97.89
6	5	4	6	10	95.10	95.10
7	11	5.5	4	50	98.53	98.47
8	6	7	6	10	95.39	95.31
9	7	4	6	50	98.09	98.18
10	10	5.5	8	10	95.57	95.63
11	2	7	4	30	98.03	98.09
12	4	7	8	30	98.09	98.11
13	15	5.5	6	30	97.93	97.89
14	3	4	8	30	98.16	98.11
15	8	7	6	50	9872	98.72

#### 3.2.2. Analysis of variance and polynomial regression

In order to treat the results statistically, an analysis of variable or ANOVA was adopted. Analysis of variance was related to one or many independent variables, which permits one to estimate whether the calculated effects are significant or obtained from noncontrolled factors; this analysis estimates a value of *p* < 0.05 [23,24]. According to *p* values (table 10), the interaction effects between Span80, D2EHPA and [MB]_0_, [MB]_0_×[MB]_0_, Span80×D2EHPA, D2EHPA×[MB]_0_ have a value of *p* < 0.05.

**Table 10.** Effects and coefficients estimated for yield response.

term	coef	SE coef	*t*	*p*
constant	97.8912	0.04689	2087.818	0.000
Span80	0.1898	0.02871	6.611	0.001
D2EHPA	0.2007	0.02871	6.990	0.001
[MB]_0_	1.6229	0.02871	56.524	0.000
(Span80)^2^	0.0722	0.04226	1.708	0.148
(D2EHPA)^2^	−0.0524	0.04226	−1.240	0.270
[MB]^2^_0_	−1.1363	0.04226	−26.887	0.000
Span80×D2EHPA	−0.1878	0.04061	−4.626	0.006
Span80× [MB]_0_	0.0830	0.04061	2.045	0.096
D2EHPA×[MB]_0_	−0.3500	0.04061	−8.619	0.000

The polynomial model of extraction yields for two units: coded and un-coded. It shows the empirical relationship between the extraction efficiency of MB and the three variables in terms of coded or un-coded forms as follows.

For the coded units:

3.5 $$\begin{array}{rl} Y(\%) & \;=97.8912+0.1898\times (\mathrm{Span}80)+0.2007\times (\text{D2EHPA})\\ & \;\;+1.6229\times ({\left[\text{MB}\right]}_{0}+0.0722\times {\left(\text{Span80}\right)}^{2}-0.0524\;\times {\left(\text{D}2\text{EHPA}\right)}^{2}\\ & \;\;-1.1363\times {\left({\left[\text{MB}\right]}_{0}\right)}^{2}-0.1878\times (\text{Span80}\times \text{D2EHPA})\\ & \;\;+0.0830\times (\text{Span}80\times {\left[\text{MB}\right]}_{0})-0.3500\times (\text{D}2\text{EHPA}\times {\left[\text{MB}\right]}_{0}). \end{array}$$

For the uncoded units:

3.6 $$\begin{array}{rl} Y\text{ (\%)} & \;=88.9162+0.0663505\times (\text{Span}80)+0.864424\times (\text{D}2\text{EHPA})\\ & \;\;+0.288869\times ({\left[\text{MB}\right]}_{0})+0.0320767\times {\left(\text{Span80}\right)}^{2}\\ & \;\;-0.0130998\times {\left(\text{D2EHPA}\right)}^{2}-0.00284083\times {{\left[\text{MB}\right]}_{0}}^{2}\\ & \;\;-0.0626156\times (\text{Span80}\times \text{D2EHPA})+0.00276827\\ & \;\;\times (\mathrm{Span}80\times {\left[\text{MB}\right]}_{0})-0.00874970\times (\text{D2EHPA}\times {\left[\text{MB}\right]}_{0}). \end{array}$$

#### 3.2.3. Response and contour surfaces

The response surfaces are used for evaluating and modelling the effects of the three variables at two levels (Span80, D2EHPA and the initial concentration of MB) on Y response which is the yield of extraction efficiency.

The study of the three-dimensional plots of response curves and contour of interaction of the D2EHPA with Span80 has been effectuated in varying the MB concentration; the results show that the highest yield with a response greater than 99.00% is in the minimum level of D2EHPA (4% by weight) and the maximum level of Span80 (7% by weight) and with the greatest initial concentration of MB (50 ppm). The dye concentration increases the removal efficiency. This may be attributed to an increase in driving force, the concentration gradient, with the increase in the initial concentration of dye [25,26].

In this case, a small amount of extractant is sufficient and the quantity of surfactant must be more important, because with high percentage of pollutant, the droplets of the internal phase become saturated and cause a decrease of the extraction efficiency and an increase of the necessary time for extraction when it is unfavourable for the emulsion stability. This agrees with previous work [27].

The study of the three-dimensional plots of the interaction of the MB with Span80 has been effectuated in varying the concentration of D2EHPA at their three levels: 4, 6 and 8. According to the graphics of contour and surface, the greatest yield Y(%) greater than 99% is localized at maximum of Span80 and maximum of MB and at minimum of D2EHPA (4%).

**Figure 7.**
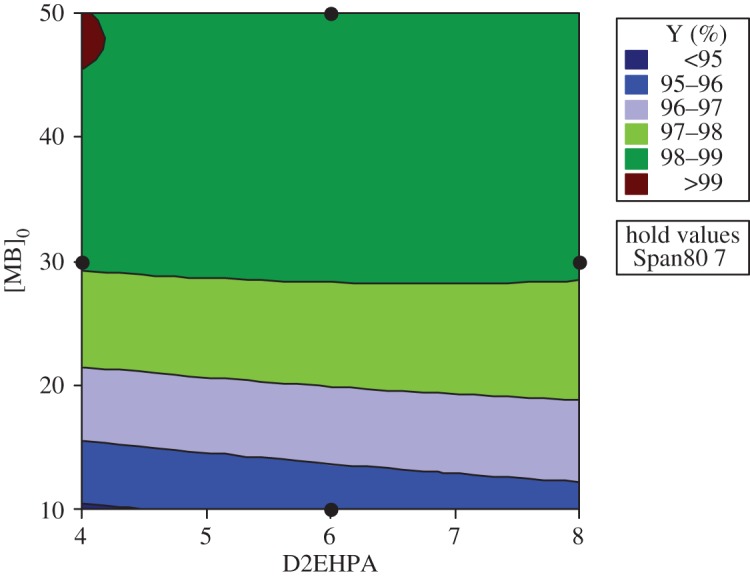
Contour plot of Y versus [BM]_0_; D2EHPA at maximum of Span80.

**Figure 8.**
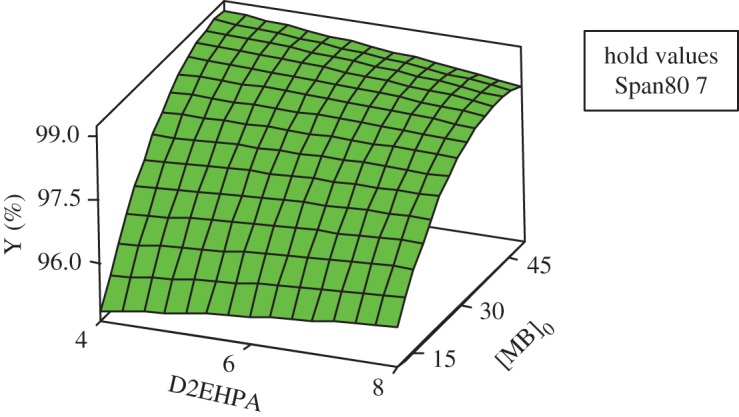
Surface plot of Y(%) versus [BM]_0_; D2EHPA at maximum of Span80.

The same interpretation for the contour and surface plots of removal percentage Y(%) about the interaction of MB concentration and D2EHPA concentration with varying Span80 concentration at their three levels, 4, 5.5 and 7, has been ascertained (figures 7 and 8).

#### 3.2.4. Optimization

The study of response surfaces and contour graphics allowed targeting the region of the experimental field where the response is the best.

The optimization results indicate the optimal values for each factor and the optimal value of extraction yield.

Obtaining a yield greater than 99% is very expensive mostly with intense and persistent dyes such as the MB; the objective was to maximize the extraction yield. A constraint was imposed on the surfactant (Span80) quantity, the extractant (D2EHPA) quantity and the initial concentration of MB. For optimization, the yield has been considered as a target, where the introduced settings were chosen as shown in table 11.

**Table 11.** Optimization of extraction of MB.

parameters	goal	lower	target	upper	weight	import
Y1 (%)	target	99.00	99.01	100	1	1

**Figure 9.**
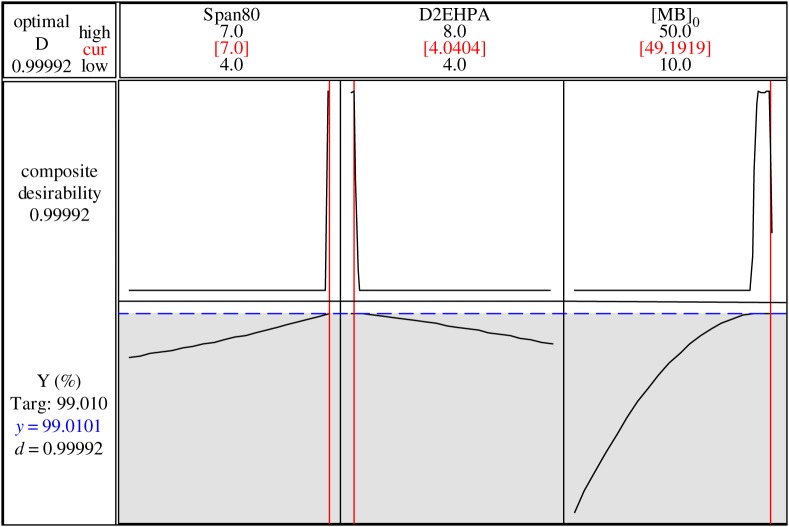
Optimization response for extraction of MB.

The optimization results of three factors are as follows: Span80: 7% by weight, D2EHPA: 4.04% by weight and MB concentration of the initial external phase: 49.19 mg l^−1^. The predicted response is equal to 99.010%, which is presented in figure 9.

While verifying the optimal results of factors, the test of MB removal gives an experimental yield of 98.70%.

## 4. Conclusion

The stability of an emulsified liquid membrane composed of Span80 as a surfactant, D2EHPA as an extractant and sulfuric acid as the internal phase was first studied according to different diluents and seven operating parameters. To find the important factors, a Plackett–Burman design was used and the influences of experimental parameters on W/O/W emulsion stability have been studied. The interest of modelling the response was to be able to calculate all responses in the domain studied without being obliged to make experiments. Different mathematical models for kerosene, gas oil, *n*-heptane and cyclohexane have been developed.

Then the removal of MB from an aqueous solution has been carried out using this emulsified liquid membrane at its stability conditions. The effects of three operating parameters (concentration of extractant, concentration of surfactant and concentration of dye), which were considered as important process parameters, were analysed from the Box–Behnken design of experiments. The modelling and the optimization of the extraction have been realized applying the response surface methodology. The results showed that the dye extraction yielding 98.72% can be achieved at optimized conditions.

Obtaining a yield greater than 99% is very expensive mostly with intense and persistent dyes such as MB; the objective was to maximize the extraction yield. A constraint was imposed on the surfactant (Span80) quantity, the extractant (D2EHPA) quantity and the initial concentration of MB. For optimization, the yield has been considered as a target, where the introduced settings were chosen.

The optimization results of three factors were as follows: Span80: 7% by weight, D2EHPA: 4.04% by weight and MB concentration of the initial external phase: 49.19 mg l^−1^. The predicted response is equal to 99.010% with a very good desirability of 0.99992.

An experimental verification of the optimum conditions was carried out and the extraction of MB was performed with a yield of 98.70%. The model was therefore adequate to represent the process.

Finally, effects of seven factors and four diluents have been studied to determine the conditions of the emulsified liquid membrane stability and 15 runs have been necessary and sufficient to optimize three parameters and to enhance the extraction yield of MB to 98.70% from an aqueous solution using this membrane at its stability conditions.

## Data accessibility

All experimental data and experimental results are presented in table 2. For the reproducibility of results, the experimental modes are well indicated in the text of the paper.
